# Characterization of the Existence of an *N*_0_-Completion of a Partial *N*_0_-Matrix with an Associated Directed Cycle

**DOI:** 10.1155/2014/835017

**Published:** 2014-02-04

**Authors:** Cristina Jordán, Juan R. Torregrosa

**Affiliations:** Instituto de Matemáticas Multidisciplinar, Universitat Politècnica de València, Camino de Vera s/n, 46022 Valencia, Spain

## Abstract

An *n* × *n* matrix is called an *N*_0_-matrix if all its specified principal minors are nonpositive. In the context of partial matrices, a partial matrix is called a partial *N*_0_-matrix if all its specified principal minors are nonpositive. In this paper we characterize the existence of an *N*_0_-matrix completion of a partial *N*_0_-matrix whose associated graph is a directed cycle.

## 1. Introduction

A *partial matrix* is a rectangular array, some of whose entries are specified while the remaining unspecified entries are free to be chosen (from a certain set). In this paper we are going to work on the set of the real numbers and to assume that all diagonal entries are prescribed. A *completion* of a partial matrix is the matrix resulting from a particular choice of values for the unspecified entries. A *completion problem* asks if we can obtain a completion of a partial matrix with some prescribed properties.

The technics to obtain this completion depend on the pattern of the partial matrix which can be *combinatorially symmetric* (i.e., *a*_*ij*_ is specified if and only if *a*_*ji*_ is) or *noncombinatorially symmetric*. Here we are going to work with this second class of partial matrices.

A natural way to describe an *n* × *n* partial matrix *A* = (*a*_*ij*_) is via a graph *G*_*A*_ = (*V*, *E*), where the set of vertices *V* is {1,2,…, *n*} and there is an arc from *i* to *j* if and only if position (*i*, *j*) of *A* is specified. In general, a directed graph (resp., nondirected graph) is associated with a noncombinatorially symmetric (resp., combinatorially symmetric) partial matrix. Since all main diagonal entries are specified we omit loops.

A *cycle* in a directed graph *G* is a sequence of arcs (*i*_1_, *i*_2_), (*i*_2_, *i*_3_), …, (*i*_*k*−1_, *i*_1_), where *i*_*k*_ ≠ *i*_*l*_ for all *k* ≠ *l*.

In the last years many completions problems have been analysed. The completion problem for partial *M*-matrices, *P*-matrices, *N*-matrices,…, has been studied by Johnson, Hogben, Urbano, Mendes,…, among others. See, for instance [1–9] and the references therein.

As a class of square real matrices that contains the *N*-matrices we define the *N*_0_ *-matrices*, *n* × *n* real matrices *A* = (*a*_*ij*_), where all its principal minors are nonpositive. Since *N*_0_-matrices are preserved by principal submatrices we define a *partial N*_0_ *-matrix* as a partial matrix whose completely specified principal submatrices are *N*_0_-matrices.

In general it is not always true that a partial *N*_0_-matrix has an *N*_0_-matrix completion as the following matrix shows (see [7]):

(1) $$\begin{array}{c} A=\left[\begin{array}{cccc} -1 & 1 & -10 & x\\ 2 & -1 & 1 & -100\\ -0.1 & 10 & -1 & 1\\ 1 & -10 & 1 & -1 \end{array}\right]. \end{array}$$

*A* is a partial *N*_0_-matrix that has an *N*_0_-matrix completion that leads us to analyze the *N*_0_-matrix completion problem depending on the pattern of the partial matrix. We have studied in [7] when a combinatorially symmetric partial *N*_0_-matrix with no null main diagonal entries such that the graph of its specified entries is a 1-chordal graph or a cycle has an *N*_0_-matrix completion.

In this paper we study the mentioned problem for partial *N*_0_-matrices that can have zeros at the main diagonal and whose associated graph is a directed cycle of length equal to the order of the matrix. In this case we may suppose without loss of generality that these matrices have the form:

(2) $$\begin{array}{c} \left[\begin{array}{ccccc} a_{11} & a_{12} & x_{13} & \cdots & x_{1n}\\ x_{21} & a_{22} & a_{23} & \cdots & x_{2n}\\ \vdots & \vdots & \vdots & & \vdots \\ x_{n-1,1} & x_{n-1,2} & x_{n-1,3} & \cdots & a_{n-1,n}\\ a_{n1} & x_{n2} & x_{n3} & \cdots & a_{nn} \end{array}\right], \end{array}$$

where *a*_*ij*_ denotes a specified entry and *x*_*ij*_ an unspecified one.

Observe that when we say “*a*_*i*,*i*+1_, *i* = 1,2,…, *n*, where the subscripts are expressed module *n*”, we are using the congruence module *n*; that is, we are considering the entries *a*_12_, *a*_23_,…, *a*_*n*−1*n*_, *a*_*n*1_.

Given a matrix *A* of size *n* × *n* the submatrix lying in rows *α* and columns *β*, *α*, *β*⊆*N* = {1,2,…, *n*} is denoted by *A*[*α* | *β*] and the principal submatrix *A*[*α* | *α*] is abbreviated to *A*[*α*].

We denote $\bar{a}=1$ if *a* = 0 and $\bar{a}=a$ if *a* ≠ 0.

In the next section we introduce necessary and sufficient conditions in order to guarantee the existence of an *N*_0_-matrix completion of a partial *N*_0_-matrix whose associated graph is a directed cycle.

## 2. Completion of Partial *N*_0_-Matrices 

It is easy to prove that *N*_0_-matrices as well as partial *N*_0_-matrices satisfy the following properties.

**Proposition 1**

Let *A* = (*a*_*ij*_) be an *n* × *n* *N*_0_-matrix.

1. *If P is a permutation matrix, then PAP* ^*T*^ *is an N*_0_ *-matrix. *
2. *If D is a positive diagonal matrix, then DA and AD are N*_0_ *-matrices. *
3. *If D is a nonsingular diagonal matrix, then DAD* ^−1^ *is an N*_0_ *-matrix. *
4. *If a*_*ii*_ ≠ 0, *i* = 1,2,…, *n, then a*_*ij*_ ≠ 0*, for all i*, *j* ∈ {1,2,…, *n*}.
5. *Every principal submatrix of* *A* *is an N*_0_ *-matrix. *

In [7] the authors proved that any *n* × *n* *N*_0_-matrix with no null diagonal entries is diagonally similar to an *N*_0_-matrix in the set:

(3) $$\begin{array}{c} S_{n}=\left\{A=\left(a_{ij}\right):\mathrm{sign}\left(a_{ij}\right)={\left(-1\right)}^{i+j+1},\;\forall i,j\right\}. \end{array}$$

But since there are *N*_0_-matrices with some entries equal to zero we need to introduce the following set:

(4) $$\begin{array}{c} wS_{n}=\left\{A=\left(a_{ij}\right):a_{ij}=0\;\text{or}\;\mathrm{sign}\left(a_{ij}\right)={\left(-1\right)}^{i+j+1},\;\;\forall i,j\right\}. \end{array}$$

We also extend the definition of *wS*_*n*_ matrices to partial matrices; that is, *P* *wS*_*n*_ consists of the *n* × *n* partial matrices *A* = (*a*_*ij*_) such that if *a*_*ij*_ ≠ 0 then sign⁡(*a*_*ij*_) = (−1)^*i*+*j*+1^, for all specified entry (*i*, *j*), *i*, *j* ∈ {1,2,…, *n*}. The following matrix *B* is an example of a matrix of *P* *wS*_4_,

(5) $$\begin{array}{c} B=\left[\begin{array}{cccc} -1 & 1 & -10 & x\\ 2 & y & 0 & -100\\ z & 10 & -1 & 1\\ 1 & -10 & 1 & -1 \end{array}\right]. \end{array}$$

The following results, consequence of Proposition 1, allow us to transform a partial *N*_0_-matrix *A* = (*a*_*ij*_), whose associated graph is a directed cycle, into a matrix whose diagonal nonzero values are −1; the nonzero elements of the first upperdiagonal are 1 and the element in position (*n*, 1) is ${\bar{a}}_{12}{\bar{a}}_{23}\cdots {\bar{a}}_{n1}$.

**Proposition 2**

Let *A* = (*a*_*ij*_) be an *n* × *n* partial *N*_0_-matrix. There exists a positive diagonal matrix *D* such that matrix *B* = *DA* = (*b*_*ij*_) is also an *N*_0_-partial matrix with *b*_*ii*_ equal to −1 or to zero, for all *i* = 1,2,…, *n*.

**Proof**

It suffices to consider *D* = {*d*_1_, *d*_2_,…, *d*_*n*_} defined *d*_*i*_ = −1/*a*_*ii*_ if *a*_*ii*_ ≠ 0 and *d*_*i*_ = 0 if *a*_*ii*_ = 0.

**Proposition 3**

Let *A* = (*a*_*ij*_) be an *n* × *n* partial *N*_0_-matrix, whose associated graph is a directed cycle, such that *a*_*ii*_ is −1 or 0, for all *i* = 1,2,…, *n*. Then there exists a diagonal matrix *D* such that *DA* *D* ^−1^ = (*c*_*ij*_) is a partial *N*_0_-matrix with *c*_*ii*_ = *a*_*ii*_ for all *i* = 1,2,…, *n*, *c*_*ii*+1_ = 1 or zero for all *i* = 1,2,…, *n* − 1 and $c_{n1}={\bar{a}}_{12}{\bar{a}}_{23}\cdots {\bar{a}}_{n1}$.

**Proof**

It suffices to consider $D=\mathrm{diag}(1,{\bar{a}}_{12},{\bar{a}}_{12}{\bar{a}}_{23},\ldots ,{\bar{a}}_{12}{\bar{a}}_{23}\cdots {\bar{a}}_{n-1n})$.

Therefore, if *A* = (*a*_*ij*_) is an *n* × *n* partial *N*_0_-matrix whose associated graph is a directed cycle, we will assume, without loss of generality, that *A* has the following structure: −1 or zeros on the main diagonal, 1's or zeros in the first upper diagonal and ${\bar{a}}_{12}{\bar{a}}_{23}\cdots {\bar{a}}_{n1}$ in position (*n*, 1).

The following theorem characterizes the *P* *wS*_*n*_ matrices as an intermediate step to obtain the desired completion. It can be easily obtained from the transformations of Propositions 2 and 3.

**Theorem 4**

Let *A* be an *n* × *n* partial matrix, *n* even (resp., odd), whose associated graph is a directed cycle. If all entries *a*_*ii*+1_, *i* = 1,2,…, *n*, where the indices are expressed module *n*, are nonzero the matrix *A* is diagonally similar to an element of *P* *wS*_*n*_ if and only if *a*_12_ *a*_23_ ⋯ *a*_*n*1_ > 0 (resp., *a*_12_ *a*_23_ ⋯ *a*_*n*1_ < 0).

Now we analyze the existence of an *N*_0_-matrix completion of a partial *N*_0_-matrix with an associated directed cycle, by distinguishing between matrices with no null main diagonal entries and matrices with some null values in the main diagonal.

**Theorem 5**

Let *A* be an *n* × *n* partial *N*_0_-matrix, with nonzero main diagonal entries such that its associated graph is a directed cycle. The following statements are equivalent:

1. *a*_12_ *a*_23_ ⋯ *a*_*n*1_ > 0 if *n* is even (*a*_12_ *a*_23_ ⋯ *a*_*n*1_ < 0 if *n* is odd),
2. *A* is diagonally similar to an element of *P* *wS*_*n*_,
3. there exists an *N*_0_-matrix completion of *A*.

**Proof**

Observe that from (4) of Proposition 1, we have that all the specified entries are nonzero. Then, from commentary after Proposition 3, we assume that all the elements in the main diagonal are −1 and the first upper diagonal is formed by 1's.

Let us suppose that *n* is even; the case *n* odd is analogous. Since the upper diagonal and the element in position (*n*, 1) are nonzero, by applying Theorem 4, the condition *a*_12_ *a*_23_ ⋯ *a*_*n*1_ > 0 is equivalent to item 2.

Now, we assume that the second statement is true. We consider *A*′ = (*a*_*ij*_′), where *a*_*ij*_′ = *a*_*ij*_ if *a*_*ij*_ is a specified value of *A*, *a*_*i*+1*i*_′ = 1 for *i* = 1,2,…, *n*, where subscripts are expressed module *n* and *a*_*n*1_′ = 1/(*a*_12_ *a*_23_ ⋯ *a*_*n*1_). Then *A*′ is an *n* × *n* partial *N*_0_-matrix, with nonzero main diagonal entries such that its associated graph is a nondirected cycle. Theorem 4.3 of [7] assures that *A*′, and therefore *A* has an *N*_0_-matrix completion.

Finally, from the note after Proposition 1, the third statement implies the second one.

Now, it arises the question about establishing an analogous result to Theorem 5, when zero entries appear in the main diagonal. The answer is negative since if we admit a zero diagonal element and a zero entry in the upper diagonal, there exist matrices in *P* *wS*_*n*_, *n* is even, such that ${\bar{a}}_{12}{\bar{a}}_{23}\cdots {\bar{a}}_{n1}$ is negative, but that admits an *N*_0_-matrix completion. For example, matrix *A* = (*a*_*ij*_) defined

(6) $$\begin{array}{c} A=\left[\begin{array}{cccc} 0 & 0 & x_{13} & x_{14}\\ x_{21} & -1 & 1 & x_{24}\\ x_{31} & x_{32} & -1 & 1\\ -1 & x_{42} & x_{43} & -1 \end{array}\right] \end{array}$$

is diagonally similar to an element of *P* *wS*_4_ by using *D* = diag⁡(−1,1, 1,1) and it has an *N*_0_-matrix completion, *A*_*c*_, although ${\bar{a}}_{12}{\bar{a}}_{23}{\bar{a}}_{34}{\bar{a}}_{41}$ is negative,

(7) $$\begin{array}{c} A_{c}=\left[\begin{array}{cccc} 0 & 0 & 0 & 0\\ 0 & -1 & 1 & -1\\ 0 & 1 & -1 & 1\\ -1 & -1 & 1 & -1 \end{array}\right]. \end{array}$$

The following results characterize this type of matrices. Note that, if there are some null main diagonal entries, the existence of an *N*_0_-matrix completion implies that if *a*_*ii*_ *a*_*i*+1*i*+1_ ≠ 0, then *a*_*ii*+1_ ≠ 0. So, we add this condition as a hypothesis. In addition, recall that we are going to assume that *a*_*ii*_ = −1 or zero for all *i* ∈ {1,2,…, *n*}; the entries in the first upper diagonal are 1 or zero and the value of the element in position (*n*, 1) is ${\bar{a}}_{12}{\bar{a}}_{23}\cdots {\bar{a}}_{n1}$.

**Theorem 6**

Let *A* be an *n* × *n*, *n* even (resp., odd), *n* ≥ 3, partial *N*_0_-matrix with some null main diagonal entries, whose associated graph is a directed cycle. Let one suppose that if *a*_*ii*_ *a*_*i*+1*i*+1_ ≠ 0 for all *i* = 1,2,…, *n*, where the indices are expressed module *n*, then *a*_*ii*+1_ ≠ 0. If there exists *a*_*ii*+1_ = 0, *i* ∈ {1,2,…, *n*}, where the indices are expressed module *n*, then there exists an *N*_0_-matrix completion.

**Proof**

Let *N*_*p*_ = {*i*_1_, *i*_2_,…, *i*_*p*_} be, with *i*_*k*_ < *i*_*k*′_ for all 1 ≤ *k* < *k*′ ≤ *p*, the corresponding indices to the negative diagonal values of matrix *A* and *N*_*l*_ = {*i*_*p*+1_,…, *i*_*p*+*l*_}, where *p* + *l* = *n* and *i*_*h*_ < *i*_*h*′_ for all *p* + 1 ≤ *h* < *h*′ ≤ *p* + *l*, the corresponding ones to the zero diagonal entries. Since there is *i*, *i* ∈ {1,2, 3,…, *n* − 1}, such that *a*_*ii*+1_ = 0, the diagonal similarity allows us to assume, without loss of generality, that *a*_*n*1_ = 1 if *p* is even and that *a*_*n*1_ = −1 if *p* is odd. Let *B* be the matrix *PAP* ^*T*^ where *P* is the permutation matrix *P* = [*e*_*i*_*p*+1__, *e*_*i*_*p*+2__,…, *e*_*i*_*p*+*l*__, *e*_*i*_1__, *e*_*i*_2__,…, *e*_*i*_*p*__], being *e*_*k*_ the canonical vector for all *k* ∈ {1,2,…, *n*}. Consider *B* partitioned in a 2 × 2 block matrix, where *B*_11_ is of size *l* × *l* and *B*_22_ is *p* × *p*.

Note that elements of the first upper diagonal *a*_*ii*+1_, *i* ∈ {1,2,…, *n* − 1}, are moved to blocks *B*_*kl*_, *k*, *l* ∈ {1,2}, depending on the value of *a*_*ii*_ and *a*_*i*+1*i*+1_: if both entries are zero, after the permutation *P* *a*_*ii*+1_ will be in *B*_11_; if *a*_*ii*_ = 0 and *a*_*ii*+1_ = 1 the element *a*_*ii*+1_ will be in *B*_12_; if *a*_*ii*_ = −1 and *a*_*ii*+1_ = 0 then *a*_*ii*+1_ will be in *B*_21_ and in other cases *a*_*ii*+1_ will be in *B*_22_. This is shown in Table 1(a). In Table 1(b) we can see the position that the element *a*_*n*1_ of *A* will occupy after the permutation.

Then we can be sure that each of the *l* first lines of the permutated matrix *B* will have as maximum a nonzero value and the last *p* ones will have exactly one −1 and only another nonzero value as maximum.

We complete with zeros the unspecified entries of blocks *B*_11_, *B*_12_, and *B*_21_. In order to complete *B*_22_ we distinguish two cases.

- The element *a*_*n*1_ of *A* is at position (*n*, *l* + 1). If *a*_*ii*_ ≠ 0 and *a*_*i*+1*i*+1_ ≠ 0, then *a*_*ii*+1_ ≠ 0 and, after the permutation, it will be in submatrix *B*_22_. If *i*_*j*_ + 1 ≠ *i*_*j*+1_ then position (*i*_*j*_, *i*_*j*+1_) of the permutated matrix *B* will be unspecified. Now we partially complete *B*_22_ as follows: *a*_1*n*_ = 1/*a*_*n*1_ and *a*_*i*_*j*_*i*_*j*_+1_ = 1 for all *j* ∈ {1,2,…, *p*}, and by Theorem 5 we get that there exists an *N*_0_-completion of *B*_22_ named *B*_22_*c*__.
- The element *a*_*n*1_ of *A* is not at position (*n*, *l* + 1). We complete *B*_22_ with 1's and −1's in order to obtain a matrix with all their diagonals formed alternatively by 1's and −1's. All the principal minors of this new matrix will be zero.

The completion of *B*, *B*_*c*_, is an *N*_0_-matrix since

- the principal minors lying rows and columns with indices in *N*_*l*_ are zero, as we can prove by developing by the nonzero elements (there is as maximum one nonzero entry by line);
- the principal minors lying rows and columns with indices in *N*_*p*_ are less than or equal to zero, since *B*_22_*c*__ is an *N*_0_-matrix completion either *a*_*n*1_ of *A* is at position (*n*, *l* + 1) or not;
- the principal minors with rows and columns with indices in *N*_*l*_ and *N*_*p*_ are zero, because of they have a row of zeros or, as before, by developing by the only nonzero element of each row, the minor is reduced to one of submatrix *B*_21_.

So, matrix *A* has an *N*_0_-matrix completion.

**Theorem 7**

Let *A* be an *n* × *n*, *n* even (resp., odd), *n* ≥ 3, partial *N*_0_-matrix with some null main diagonal entries, whose associated graph is a directed cycle. Let one suppose that if *a*_*ii*_ *a*_*i*+1*i*+1_ ≠ 0 for all *i* = 1,2,…, *n* where the indices are expressed module *n*, then *a*_*ii*+1_ ≠ 0. If *a*_*ii*+1_ ≠ 0 for all *i* ∈ {1,2,…, *n*}, where the indices are expressed module *n*, then the condition *a*_12_ *a*_23_ ⋯ *a*_*n*1_ > 0 for *n* even (resp., *a*_12_ *a*_23_ … *a*_*n*1_ < 0 for *n* odd) is necessary and sufficient for the existence of an *N*_0_-matrix completion.

**Proof**

Let *n* be even. If *a*_12_ *a*_23_ … *a*_*n*1_ > 0 some changes in the proof of the above theorem gives the desired completion. Specifically, choose the zero diagonal entry *a*_*kk*_ with less index. If 1 ≤ *k* ≤ *n* − 1 since *a*_12_ *a*_23_ ⋯ *a*_*n*1_ > 0 matrix *A* can be transformed by diagonal similarity in a matrix such that if *p* is even, *a*_*n*1_ = 1 and *a*_*kk*+1_ are equal to a positive value and if *p* is odd, *a*_*n*1_ = −1 and *a*_*kk*+1_ are equal to a negative value. By permutation similarity we get a block-matrix analogous to the previous result and by a similar reasoning we get that there exists an *N*_0_-matrix completion of *A*. If *k* = *n* we transform *A* by the permutation *P* = [*e*_*n*_, *e*_1_,…, *e*_*n*−2_, *e*_*n*−1_], where *e*_*k*_ is the canonical vector for all *k* ∈ {1,2,…, *n*} and we proceed in a similar way to the previous result.

Now, we are going to prove the necessity of the condition by induction on *n*; that is, if there exists an *N*_0_-completion of *A*, *A*_*c*_ = (*c*_*ij*_), then *c*_12_ *c*_23_ ⋯ *c*_*n*1_ > 0 if *n* is even and *c*_12_ *c*_23_ ⋯ *c*_*n*1_ < 0 if *n* is odd. We denote *α* = *c*_12_ *c*_23_ ⋯ *c*_*n*1_.

For *n* = 3, if we suppose that *α* > 0 by analyzing the seven different cases that arise depending on the number of zeros, one, two, or three in the main diagonal, we get that det⁡*A*_*c*_ > 0, that is a contradiction. Then *α* < 0.

Let *n* > 3 be. Suppose *n* is even (if *n* is odd the process is analogous). We are showing that in the mentioned conditions if there exists an *N*_0_-completion of *A*, then *α* > 0. Let *A*_*c*_ be an *N*_0_-completion of *A*.

Let us suppose by hypothesis of induction (HI) that the statement is true for all 3 ≤ *k* ≤ *n* − 1. Since *A*_*c*_ is an *N*_0_-matrix, we obtain, from the 2 × 2 principal minors, that all the entries of the first under diagonal are greater than or equal to zero; that is, *c*_*i*−1*i*_ ≥ 0 for all *i* ∈ {1,2,…, *n*}.

In addition, if we consider det⁡*A*[{*i*, *i* + 1,…, *i* + *p*}] with *i* ∈ {1,2,…, *n* − *p*}, *p* ∈ {2,…, *n* − 2}, we get that *c*_*ij*_ > 0 if *i* − *j* is odd and *c*_*ij*_ < 0 if *i* − *j* is even for *i* ∈ {1,2,…, *n* − 1}, *j* ∈ {1,2,…, *n* − 2}, *i* > *j*.

From the nonpositivity of det⁡*A*[{*i*, *j*}] for all *i* ∈ {1,2,…, *n* − 2}, *j* ∈ {3,…, *n*}, we obtain that the upper diagonals follow the same rule of signs of the under diagonals, alternatively positive and negative, but with the option of zero; that is, *c*_*ij*_ ≥ 0 if *j* − *i* is odd and *c*_*ij*_ ≤ 0 if *j* − *i* is even for *i* ∈ {1,2,…, *n* − 2}, *j* ∈ {3,…, *n*}, *i* < *j*.

Now we study the case in which there exists *i* ∈ {3,…, *n*} such that *a*_1*i*_ ≠ 0.

- If *i* ∈ {3,…, *n* − 1} we consider det⁡*A*[{1, *i*, *i* + 1,…, *n*}]. The (*n* − *i* + 2)×(*n* − *i* + 2) submatrix of *A*_*c*_, *A*′ = *A*_*c*_[{1, *i*, *i* + 1,…, *n*}] can be considered as a completion of a partial *N*_0_ matrix of size strictly smaller than *n* with all the first upper diagonal formed by 1's. If *A*′ has at least a zero diagonal entry, taking into account that *c*_1*i*_ ≤ 0 if *i* is odd and *c*_1*i*_ ≥ 0 if *i* is even, the hypothesis of induction allows us to assure that the entry in position (*n* − *i* + 2,1) of *A*′; that is, *α* is positive. In the other case, if all the diagonal entries are nonzero we get the same conclusion by Theorem 5. This ends the proof in this case.
- If *i* = *n* and *c*_1*i*_ = 0 with *i* ∈ {1,…, *n* − 1}, we analyze two cases: (b.1) *c*_11_ = 0 or *c*_*nn*_ = 0 and (b.2) *c*_11_ ≠ 0 and *c*_*nn*_ ≠ 0. In the first one, we get *α* > 0 from det⁡*A*_*c*_[{1,3, *n*}]. If *c*_11_ ≠ 0, *c*_*nn*_ ≠ 0 and *c*_22_ = 0 and *c*_*ii*_ ≠ 0 for all *i* ∈ {3,…, *n* − 1} we get *α* > 0 from det⁡*A*_*c*_[{1, *n* − 1, *n*}] ≤ 0. If *c*_11_ ≠ 0, *c*_*nn*_ ≠ 0 and there exists *i* ∈ {3,…, *n* − 1} such that *c*_*ii*_ = 0 we get also the same result from det⁡*A*_*c*_[{1, *i*, *n*}] ≤ 0.

In this case *c*_1*i*_ = 0 for all *i* ∈ {3,…, *n*} if some entry *c*_*ij*_ of the upper triangular part of *A*_*c*_ is nonzero, we also obtain that *α* > 0 by using det⁡*A*_*c*_[{1,2,…, *i* − 1, *i*, *j*, *j* + 1,…, *n*}] ≤ 0 and HI.

Therefore, it remains to analyze the case in which all the upper triangular part except the first upper diagonal is zero. As we will see now, most of the cases can not be given.

Let *c*_*ii*_ be the nonzero diagonal entry with less index. If *i* ≥ 5 from det⁡*A*_*c*_[{1,2, 3, *i*}] ≤ 0 or if *i* ∈ {1,2, 3,4} and *n* ≥ *i* + 4 from det⁡*A*_*c*_[{1, *n* − 2, *n* − 1, *n*}] ≤ 0 we get a contradiction. Now we study the remaining cases depending on the values of *n* and *i*.

If *n* = 4 from det⁡*A*_*c*_ ≤ 0 we get *α* > 0 (if *i* = 4 to show it we also use det⁡*A*_*c*_[{1,2, 4}] ≤ 0).

If *n* = 5 and *c*_*i*+1*i*+1_ = 0 from det⁡*A*_*c*_ ≤ 0 we obtain *α* < 0 (if *i* = 3 to show it we also use det⁡*A*_*c*_[{1,2, 4,5}] ≤ 0 and det⁡*A*_*c*_[{1,2, 4}] ≤ 0 if *i* = 4). If *n* = 5, *c*_*i*+1*i*+1_ ≠ 0 and *i* ∈ {2,3} from det⁡*A*_*c*_ ≤ 0 we get *α* < 0 (if *i* = 2 to show it we also use det⁡*A*_*c*_[{2,4, 5}] ≤ 0 and det⁡*A*_*c*_[{1,2, 4}] ≤ 0 if *i* = 3); if *i* = 4det⁡*A*_*c*_[{1,2, 4}] ≤ 0 and det⁡*A*_*c*_[{1,2, 3,5}] ≤ 0 leads a contradiction.

If *n* = 6 and *i* = 3 from det⁡*A*_*c*_ ≤ 0 we get *α* > 0 (if *c*_*i*+1*i*+1_ = 0 to show it we also use det⁡*A*_*c*_[{1,2, 5,6}] ≤ 0 and det⁡*A*_*c*_[{1,2, 4,5, 6}] ≤ 0 and if *c*_*i*+1*i*+1_ ≠ 0, the nonpositivity of det⁡*A*_*c*_[{1,2, 4}]. If *n* = 6, *i* = 4 and *c*_*i*+1*i*+1_ = 0 from det⁡*A*_*c*_ ≤ 0 and det⁡*A*_*c*_[{1,2, 4}] ≤ 0 we get *α* > 0. In this case if *c*_*i*+1*i*+1_ ≠ 0 the nonpositivity of det⁡*A*_*c*_[{1,2, 4}] and det⁡*A*_*c*_[{1,2, 3,5}] leads to a contradiction.

Finally, if *n* = 7 and *i* = 4 we get a contradiction by using det⁡*A*_*c*_[{1,2, 4}] ≤ 0 and also det⁡*A*_*c*_[{1,2, 3,5}] ≤ 0 if *c*_*i*+1*i*+1_ ≠ 0, or if *c*_*i*+1*i*+1_ = 0 the nonpositivity of det⁡*A*_*c*_[{1,2, 3,5, 6}] and det⁡*A*_*c*_[{1,2, 3,5, 6,7}].

We sum up the results of Theorems 6 and 7 in Table 2. One can consider a similar one for *n* odd.

## Figures and Tables

**Table:** (a)

**a**_**ii**+1_	*a*_*i*+1*i*+1_ = 0	*a*_*i*+1*i*+1_ ≠ 0
*a*_*ii*_ = 0	*B*_11_	*B*_12_
*a*_*ii*_ ≠ 0	*B*_21_	*B*_22_

**Table:** (b)

**a**_**n**1_	*a*_11_ = 0	*a*_11_ ≠ 0
*a*_*nn*_ = 0	(*l*, 1) (in *B*_11_)	(*l*, *l* + 1) (in *B*_12_)
*a*_*nn*_ ≠ 0	(*n*, 1) (in *B*_21_)	(*n*, *l* + 1) (in *B*_22_)

**Table 2:** *Under the assumption that *a*_*ii*_ *a*_*i*+1*i*+1_ ≠ 0 implies *a*_*ii*+1_ ≠ 0.

	∀*i* *a*_*ii*+1_ ≠ 0	∃*i* *a*_*ii*+1_ = 0*
∀*i* *a*_*ii*_ ≠ 0	*a*_12_ *a*_23_ … *a*_*n*1_ > 0 iff ∃*N*_0_-matrix completion (Theorem 5)	*N*_0_-matrix completion has no sense (Proposition 1)
∃*i* *a*_*ii*_ = 0	*a*_12_ *a*_23_ … *a*_*n*1_ > 0 iff ∃*N*_0_-matrix completion (Theorem 7)	∃*N*_0_-matrix completion (Theorem 6)

*A* is an *n* × *n*, **n** **even**, partial *N*_0_-matrix, whose associated graph is a directed cycle.
